# A Small Molecule Inhibitor of CTP Synthetase Identified by Differential Activity on a *Bacillus subtilis* Mutant Deficient in Class A Penicillin-Binding Proteins

**DOI:** 10.3389/fmicb.2020.02001

**Published:** 2020-08-26

**Authors:** Kaveh Emami, Ling Juan Wu, Jeff Errington

**Affiliations:** Centre for Bacterial Cell Biology, Biosciences Institute, Newcastle University, Newcastle upon Tyne, United Kingdom

**Keywords:** CTP synthetase, *pyrG*, isoquinoline antibiotic, *Bacillus subtilis*, class A penicillin-binding protein

## Abstract

In the course of screening for compounds with differential growth inhibition activity on a mutant of *Bacillus subtilis* lacking all four class A penicillin-binding proteins (*Δ4*), we came across an isoquinoline derivative, IQ-1 carboxylic acid (IQC) with relatively high activity on the mutant compared to the wild type strain. Treated cells were slightly elongated and had altered chromosome morphology. Mutants of *Δ4* resistant to IQC were isolated and subjected to whole genome sequencing. Most of the mutants were affected in the gene, *pyrG*, encoding CTP synthetase (CTPS). Purified wild type CTPS was inhibited *in vitro* by IQC. Two of the three mutant proteins purified showed decreased sensitivity to IQC *in vitro*. Finally, inhibition by IQC was rescued by addition of cytidine but not uridine to the growth medium, consistent with the notion that IQC acts by reducing the synthesis of CTP or a related compound. IQC provides a promising new starting point for antibiotic inhibitors of CTPS.

## Introduction

The continuous rise in resistance to antibiotics is encouraging the search for antibiotics with new modes of action. Historically, the cell wall has been one of the most productive targets for discovery and development of antibiotics, because it is usually essential for bacterial growth and viability and there is no equivalent of the structure or synthetic pathway in human cells. The main essential component of virtually all bacterial cell walls is peptidoglycan (PG). Synthesis of PG requires one or more enzymes with glycosyltransferase (GTase) activity. It recently emerged that the RodA and FtsW “SEDS” enzymes are the main, essential GTases in many bacteria (Cho et al., 2016; Meeske et al., 2016; Emami et al., 2017; Sjodt et al., 2018) and thus are interesting potential targets for novel antibiotics.

The SEDS proteins are partially redundant to class A penicillin-binding proteins (PBPs), which also have PG glycosyltransferase activity (Sauvage et al., 2008). We previously described a screen for RodA inhibitors looking for chemical activities that were more potent on a strain of *Bacillus subtilis* deleted for all four known class A PBPs (Mcpherson and Popham, 2003) than on wild type cells, based on the idea that this *Δ4* mutant should rely completely on RodA for PG polymerization during cell growth (Emami et al., 2017). Here we describe the finding and characterization of isoquinoline compounds that have the required specificity for the *Δ4* mutant over wild type cells. However, the compounds turn out to target not RodA, but rather an enzyme CTP synthetase encoded by *pyrG*, required for synthesis of cytidine triphosphate (CTP). The compound differs from known CTP synthetase inhibitors in lacking similarity to the enzyme substrate or products. The compound may provide a starting point for the development of a new family of anti-infectives.

## Results

### Isoquinoline 1-Carboxylic Acid Has Higher Antibiotic Activity on *B. subtilis* Lacking All Four Class A Penicillin-Binding Proteins

**FIGURE 1:**
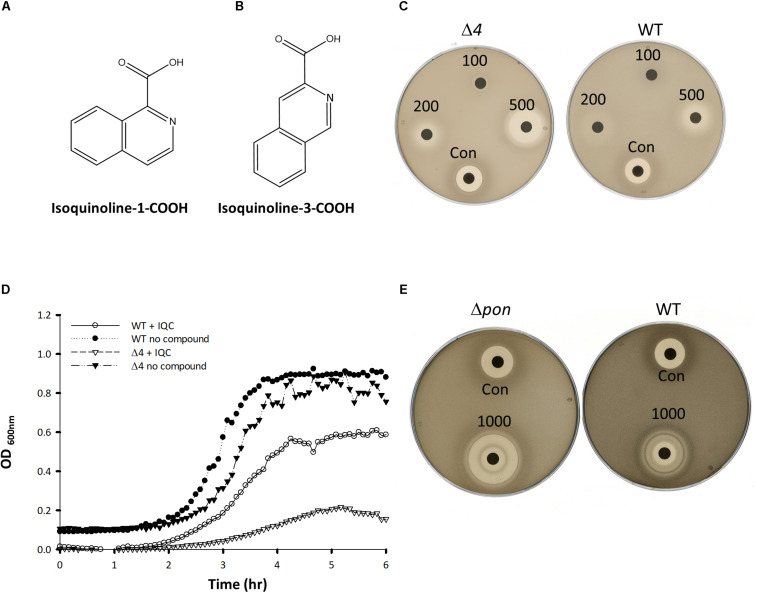
Compound structures and effects on bacterial growth. **(A,B)** The structures of Isoquinoline-1-carboxylic acid **(A)**, and Isoquinoline-3-carboxylic acid **(B)**. **(C,E)** Disc assays showing the activity of IQC against the wild type (WT) and the Δ*4* **(C)** and Δ*ponA* **(E)** mutants. The radius of the inhibition zones for *Δ4* at 500 μg was 7 vs. 2 mm for the WT. In Panel **(C)** the values were 10 and 3 mm for the Δ*ponA* and WT strains (1000 μg disc), respectively. Numbers refer to the amounts of IQC added to the discs (μg). CON, Citrox (5 μL). **(D)** Effect of IQC (40 μg mL^–1^) on growth of wild type and mutant strains in liquid culture.

We previously described a chemical genetics screen for antibiotic compounds potentially targeted the RodA GTase, based on differential activity on a *B. subtilis* mutant lacking all 4 class A PBPs (called *Δ4*) vs. the wild type, 168ca (Emami et al., 2017). Culture supernatants from an actinomycete strain, DEM20654, were found to inhibit the growth of the *Δ4* strain much more than the isogenic wild type strain. In the course of attempting to purify the active component from the complex mixture of metabolites produced by DEM20654 using HPLC-mass spectrometry, we frequently observed the presence of a molecule with a monoisotopic mass of 129.057, potentially a fragment of the active species, or an intermediate in its synthesis. A search of the Dictionary of Natural Products highlighted quinoline and isoquinoline as the only plausible compounds with that accurate mass. We obtained several commercially available compounds with a quinoline or isoquinoline nucleus, as well as various other similar compounds and tested them for antibiotic activity by a disc diffusion assay on the wild type and *Δ4* strains. Two closely related compounds, isoquinoline-1-carboxylic acid (hereafter IQC; Figure 1A) and isoquinoline-3-carboxylic acid (IQC3; Figure 1B), were found to have antibiotic activity that was greater on the *Δ4* mutant than on the wild type (Figure 1C). The other compounds tested were mainly inactive or showed no signs of differential activity on the two strains (Table 1 and Supplementary Figure S1). Subsequent studies were mainly conducted with IQC, which has a minimum inhibitory concentration (MIC) of 300 μg mL^–1^ for the WT (168ca) and 50 μg mL^–1^ for *Δ4*. Figure 1D shows the results of a liquid culture experiment in which 40 μg mL^–1^ IQC almost completely inhibited the growth of the *Δ4* mutant but had a much lesser effect on wild type cells. In some of the experiments described below we replaced *Δ4* with a mutant (*ΔponA*) deficient only in the major class A PBP, PBP1. This single mutant also showed differential sensitivity to the compound relative to wild type (168ca) cells (Figure 1E) and had a similar MIC (50 μg mL^–1^) to the *Δ4* mutant.

**TABLE 1:** Testing various compounds for differential antibiotic activity on the *Δ4* and WT strains.

**Compound**	**Inhibition zone (mm)**	**Differential activity**
Isoquinoline	1	No
Isoquinoline-1-Carboxylic acid	10	Yes
Isoquinoline-3-Carboxylic acid	14	Yes
Isoquinoline-4-Carboxylic acid	ND	
Isoquinoline-5-Carboxylic acid	ND	
Quinoline	ND	
Quinoline-3-carboxylic acid	1	No
Quinoline-4-carboxylic acid	ND	
3-Methylquinoline	ND	
3-methyl-quinoline-4-carboxylic acid	ND	
Quinoxaline	ND	
Quinaldic acid	8	No
Chloroquine phosphate	ND	
2-amino-3H-quinozaline-4-one	ND	
NSC308884*	5	No

*5-Amino-2-(2-(diethylamino)ethyl-1-4-benzo[de]Isoquinoline-1,3(2H)-dione. Approximately 500 μg of each compound was added to 6 mm discs. The inhibition zone assay results were from *Δ4* strain plates. ND: not detected.

### Mode of Action Studies Suggest an Effect on Chromosome Dynamics

**FIGURE 2:**
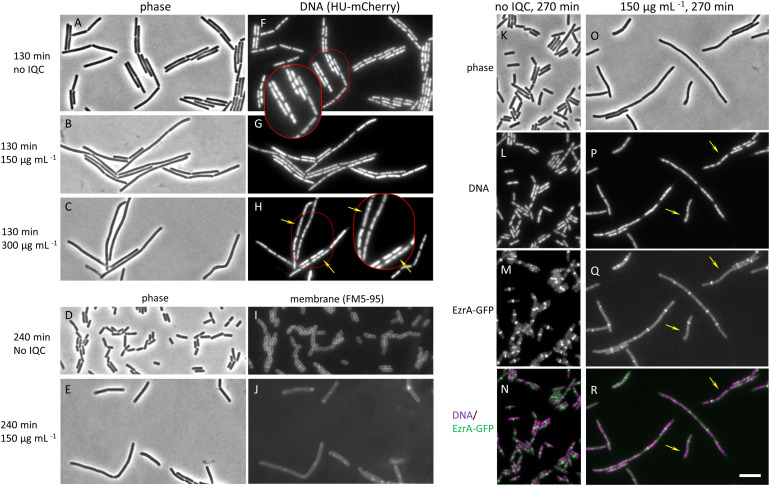
Effects of IQC on cell morphology. **(A–C)** and **(F–H)** Effects of different concentrations of IQC on *B. subtilis* cells. The wild type strain 168ca, carrying an *ezrA-gfp* fusion (native promoter at native locus) and an Hbsu-mCherry fusion (at *amyE*; for labeling DNA), was grown in the presence of IQC or 0.01% DMSO at 30°C for 130 min. **(A–C)** phase contrast images. **(F–H)** images of Hbsu-mCherry showing the nucleoids. **(A,F)** no IQC (0.01% DMSO). **(B,G)** 150 μg mL^–1^ IQC. **(C,H)** 300 μg mL^–1^. Arrows in H point to elongated nucleoids. **(D,E,I,J)** Effect of IQC treatment on cell length. Wild type strain 168ca grown in LB was treated with 150 μg mL^–1^ IQC for 240 min at 30°C. Membranes were stained with membrane dye FM5-95. **(D,E)** Phase contrast images. **(I,J)** images of membranes. **(K–R)** Effect of IQC treatment on the localization of early cell division protein EzrA. The wild type strain 168ca, carrying an *ezrA-gfp* fusion (native promoter at native locus) and an Hbsu-mCherry fusion (at *amyE*; for labeling DNA), was grown in the presence of IQC **(O–R)** or DMSO **(K–N)** at 30°C for 270 min. **(K,O)** Phase contrast images. **(L,P)** Images of Hbsu-mCherry showing the nucleoids. **(M,Q)** Images of EzrA-GFP used as a marker for the localization of cell division protein FtsZ. **(N,R)** Overlay of Hbsu-mCherry and the EzrA-GFP images. EzrA-GFP is shown in green and DNA in magenta. Arrows point to locations where EzrA localization is perturbed or absent and chromosome are not properly segregated. The inserts show the enlarged images of the cells circled in red in panels **(F,H)**.

If IQC acted on RodA and or FtsW we expected to see a disruption of cell growth, rod shape and or division in both wild type and the *Δ4* or *ΔponA* mutant strains, with the effects being more severe in the mutants than in the wild type (because the class A PBPs remain active in the wild type). We therefore used phase contrast and fluorescence microscopy to examine cells of each of the strains, in the presence and absence of IQC. Although the wild type was more resistant to the compound, the phenotypic effects of IQC treatment turned out to be similar in all three of the strains (Supplementary Figure S2). Because the *Δ4* and *ΔponA* strains already have a slight morphological phenotype (Popham and Setlow, 1996) we focused our attention on the wild type. As shown in Figure 2, treatment with IQC caused an increase in cell length, as judged both by phase contrast (A-E) and fluorescence microscopy (membrane stain; I, J), consistent with an inhibition of cell division. However, examination of chromosome morphology in cells bearing an *hbsU-GFP* fusion, which uniformly labels the entire nucleoid (Kohler and Marahiel, 1997) revealed impaired chromosome segregation and or organization in IQC treated cells (Figures 2F–H). The yellow arrows point to nucleoids that are highly elongated compared to the relatively compact nucleoids in the untreated cells (panel F).

Since perturbations of chromosome dynamics are well known to impact on cell division, and specifically on the assembly and localization of the master regulator of cell division, FtsZ, whereas RodA / FtsW inhibition should act on PG synthesis downstream of FtsZ ring assembly (Adams and Errington, 2009), we examined FtsZ localization in the treated cells using EzrA-GFP as a surrogate, functional marker for FtsZ ring formation (Levin et al., 1999; Gamba et al., 2009). As shown in Figures 2K–N, the short untreated cells virtually all had an EzrA-GFP band very close to the mid-point of the rod. However, GFP bands were much less frequent per unit length in the elongated treated cells. Furthermore, the elongated nucleoids in treated cells corresponded to the locations where expected EzrA rings were absent (yellow arrows in panels Q – R point to sites where bands would have been expected), showing that division inhibition occurred early and that it might be a result of defects in chromosome organization. Supplementary Figure S2 shows that similar phenotypic effects were seen for the *Δ4* and *ΔponA* mutants. These results suggested that the primary effect of IQC might be on chromosome dynamics, rather than on FtsZ or one or more of the SEDS proteins.

To investigate the mode of action further, we tested the effects of IQC on a collection of strains with *lacZ* reporter genes coupled to promoters sensitive to various antibiotic challenges. Supplementary Figure S3 shows that IQC triggered expression of a DNA damage reporter construct based on a late promoter of the Φ105 prophage, and of *liaI-lacZ*, which is thought to respond generally to cell envelope damage (Radeck et al., 2017). None of the other reporter strains tested showed a response, including those sensitive to inhibition of protein synthesis, transcription, or fatty acid synthesis (Hutter et al., 2004). Isoquinoline-3-carboxylic acid (but not picolinic acid, which has the carboxylic substituted pyridind ring and no benzyl ring) triggered the same two reporters, supporting the notion that this compound has a similar mode of action to IQC.

### IQC-Resistance Mutations Mainly Map to the *pyrG* (CTP Synthetase) Locus

**FIGURE 3:**
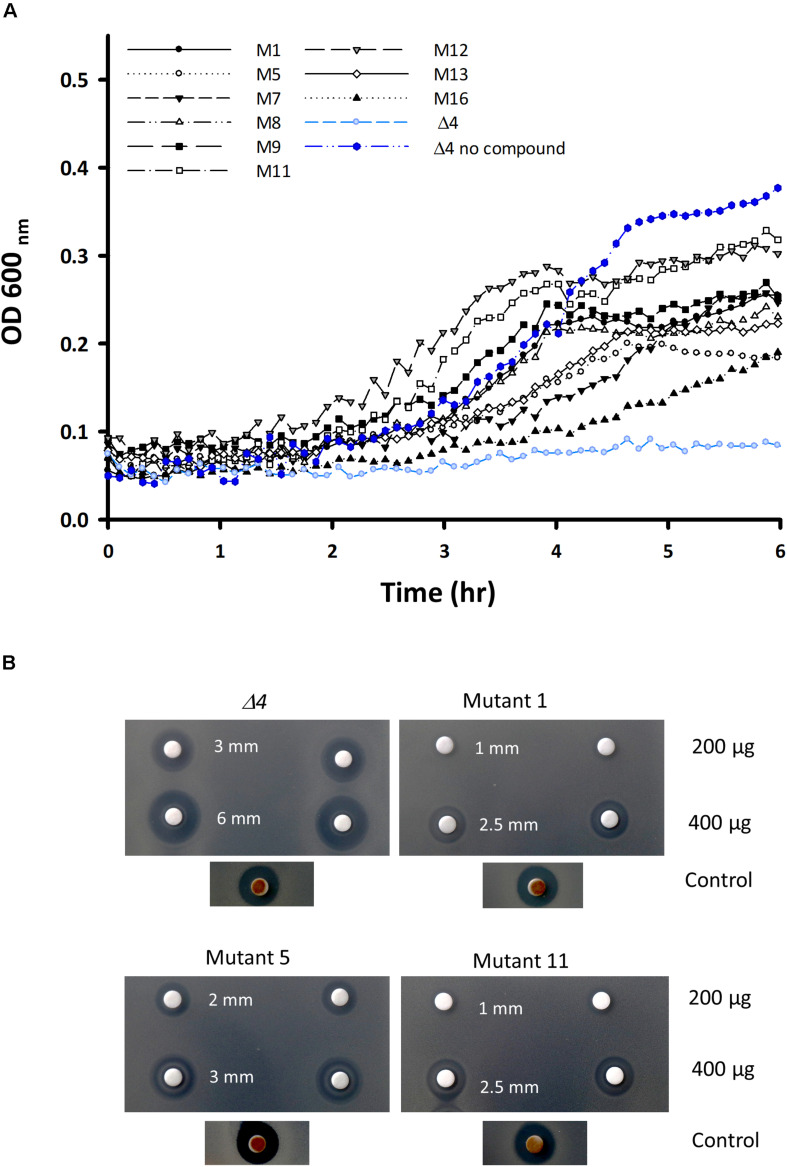
Characterization of the mutants of the *Δ4* strain resistant to IQC. **(A)** Growth curves of *Δ4*, and various resistant mutants derived from *Δ4*, in the presence of an inhibitory concentration of IQC (100 μg ml^–1^). **(B)** Disc diffusion assay comparing the sensitivities of the 3 main *pyrG* mutants (mutants # 1, 5, and 11) and the *Δ4* parental strain to 200 and 400 μg IQC in duplicate. The positive control disc contained 5 μL of Citrox.

To gain further insights into the mode of action of IQC we isolated a series of mutants resistant to the compound. We took the *Δ4* mutant strain and plated it in the presence of 300 μg ml^–1^ IQC (∼6× MIC) to select for resistant mutants, which emerged at a frequency of approximately 1×10^–7^ per cell. Figure 3A shows examples of the growth of wild type, parental (*Δ4*) strain and several resistant mutant strains cultured in the presence of 100 μg ml^–1^ IQC. This concentration of IQC abolished growth of the parent *Δ4* strain but all of the mutants showed increased growth rate; most showed similar level of growth to the parent strain with no compound. Figure 3B demonstrates the resistance of the *pyrG* mutants relative to the parental *Δ4* strain based on disc diffusion assays. Killing by 200 or 400 μg IQC on discs gave broader zones of growth inhibition on the parent *Δ4* strain than on the mutants, whereas the control compound gave equal sized zones of clearing on both *Δ4* and the mutants.

Having verified that the isolates were indeed resistant to IQC we used whole genome sequencing to identify the site(s) of the mutations. As summarized in Table 2, mutations likely to be responsible for resistance in 9 different mutants were identified. In all but 2 we found single point mutations in the *pyrG* gene, all of which would result in single amino acid substitutions. The other two mutants both had a mutation in the *rpoB* gene, encoding the β-subunit of RNA polymerase, together with additional mutations in *rpoC* or *greA*. All 4 of these mutations seemed likely to affect general transcription, so we focused on the *pyrG* mutations. *pyrG* encodes CTP synthetase (CTPS), an essential enzyme required to make CTP, using UTP, glutamine and ATP as substrates (Mccluskey et al., 2016).

**TABLE 2:** Locations of the IQC-resistance mutations and predicted effects and disc diffusion assay results.

**Mutant**	**Gene**	**aa substitution**	**Nucleotide**	Inhibition zone radius** mm
1	*pyrG*	E156 > G	467	4
5,8,9,13*	*pyrG*	P485 > L	1454	5, 5.5, 5, 5.5
11	*pyrG*	R159 > C	476	4
12	*pyrG*	S201 > I	602	4
7	*rpoB*	V1059 > F	3175	7
	*rpoC*	R326 > L	977	
16	*rpoB*	A621 > E	1922	4
	*greA*	K120 > E	148	

*Mutant 13 has an additional silent mutation in the *skiZ* gene (aa 113 unchanged, nucleotide # in *skiZ*: 339). **Inhibition zone was measured from the external edge of the paper disc with 500 μg IQC. In the same experiment the inhibition zone for *Δ4*, Δ*ponA*, and 168ca (WT) were 11, 10, and 5 mm, respectively.

Table 2 shows the inhibition zones of different mutants in response to IQC by disc diffusion assay. In general, the *pyrG* mutation at position 1454 (# 5, 8, 9, and13) resulted in a higher level of resistance to IQC, becoming more or less similar to the WT (168ca), and the other *pyrG* mutations (# 1, 11, and 12) resulted in resistance even higher than the WT. The MIC values for the *pyrG* mutants were estimated to be 250 for mutant #5 and 400 μg mL^–1^ for mutants #1 and #11.

To confirm that the mutations identified actually conferred resistance to IQC, we PCR amplified the *pyrG* region from three of the resistant mutants (# 1, 5, 11) and also the parental strain *Δ*4 (as a control), and transformed the PCR products into the sensitive parental strain (*Δ4*), with selection for growth on IQC. Resistant colonies were readily obtained for DNA samples from each of the resistant mutants but not the wild type control DNA. We then PCR amplified and sequenced the *pyrG* genes from one transformant each, and confirmed that in each case they contained the same substitution as in the donor of the mutant DNA.

### IQC Inhibits CTP Synthetase *in vitro*

The finding of resistance mutations in the *pyrG* gene suggested that IQC is an inhibitor of CTPS and that the resistance mutations alter the properties of the protein to overcome IQC action. To test this, we overexpressed and purified the wild type CTPS and several of the resistant mutant proteins. CTPS catalyses the following reaction.

ATP+H⁢O2+L-glutamine+UTP=ADP+CTP+2H++L-glutamate+phosphate

**FIGURE 4:**
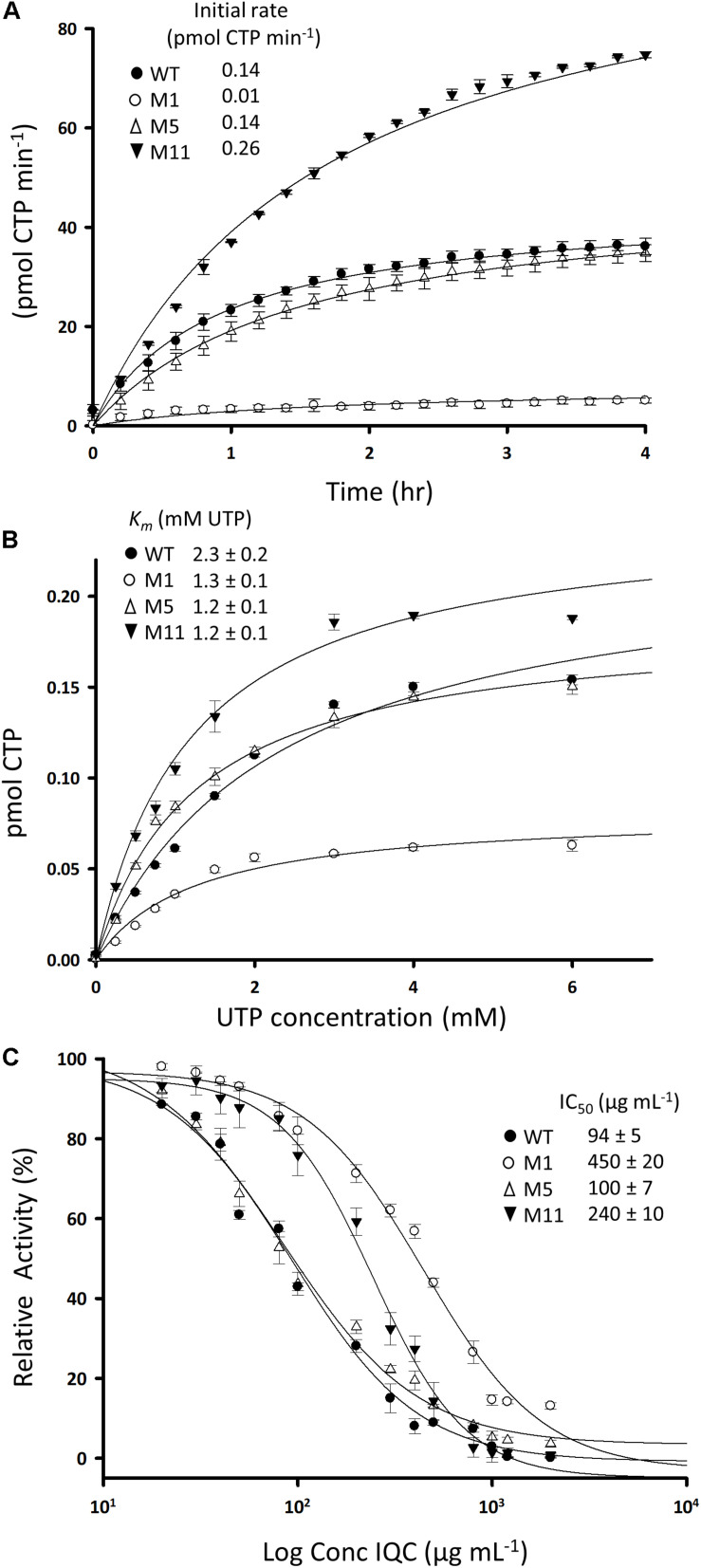
Activities of wild type and mutant CTPS enzymes and the effects of inhibition by IQC. **(A)** Reaction velocities for the wild type and mutant enzymes. Production of CTP from reactions containing 2 mM UTP was measured by absorbance at 291 nm in the presence of ATP, L-glutamine, and Mg^2+^. **(B)** Relationship between UTP concentration and reaction velocity in assays equivalent to **(A)**. Inset shows calculated values for *K*_*m*_. **(C)** Dose response curves for inhibition of the wild type and mutant enzymes by IQC. Inset shows calculated IC_50_ values. The reactions were carried out in triplicate and the data points are the mean of three replicates. Errors are ± standard deviation. The fit curves and the IC_50_ values were calculated using SigmaPlot 13.1.

We set up an activity assay based on spectrophotometric detection of CTP production in the presence of UTP, ATP and glutamine. The time course for a typical reaction with the wild type enzyme and measurement of the apparent *K*_*m*_ are shown in Figures 4A,B, with summaries in Table 3. We then examined several of the mutant enzymes. One of the mutants (#11, R159C) exhibited a substantially faster initial rate than the wild type, whereas #1, E156G, was much less active (Figure 4A). Surprisingly, mutant #5 (P485L) was virtually indistinguishable from the wild type enzyme. The mutant enzymes did not differ appreciably from the wild type in terms of affinity, although their *K*_*m*_ values were all slightly lower (Figure 4B and Table 3). Unfortunately, we could not use this assay to examine the effects of IQC on enzyme activity because the compound absorbed strongly at the wavelength used to measure CTP production (291 nm). We therefore employed an alternative assay based on the conversion of glutamine to glutamate. As shown in Figure 4C, IQC inhibited wild type CTPS activity in a dose responsive manner, with an IC_50_ of approximately 94 μg mL^–1^. Mutant #5, again, somewhat surprisingly, had only a slightly higher level of resistance to the compound (∼100 μg mL^–1^), so these experiments did not shed light on the ability of the strains containing this mutation to resist IQC *in vivo*. However, both mutants #1 and #11 had substantially higher IC_50_ values, of 450 and 240 μg mL^–1^, respectively, despite the fact that they differed so much in catalytic velocity (#11 higher and #1 lower than wild type).

**TABLE 3:** Characterization of the wild type and mutant CTPS enzymes.

**Enzyme**	**Initial**	***K*_*m*_**	**V_max_**	**IQC IC_50_**
	**rate**	**(mM UTP)**	**(pmol CTP min^–1^)**	**(μg mL^–1^)**
WT	0.14	2.3 ± 0.19	0.23 ± 0.01	94 ± 5
Mutant 1	0.01	1.2 ± 0.13	0.08 ± 0.00	450 ± 20
Mutant 5	0.14	1.2 ± 0.07	0.19 ± 0.00	100 ± 7
Mutant 11	0.26	1.2 ± 0.12	0.25 ± 0.01	240 ± 10

Similar conclusions in relation to the relative activities of the mutant and the wild type proteins, and inhibition by IQC, were obtained by qualitative assays based on MALDI-TOF discrimination of CTP / UTP and glutamine / glutamate (Supplementary Figure S4).

### Growth of IQC-Treated Cells Is Partially Rescued by Exogenous Cytidine

**FIGURE 5:**
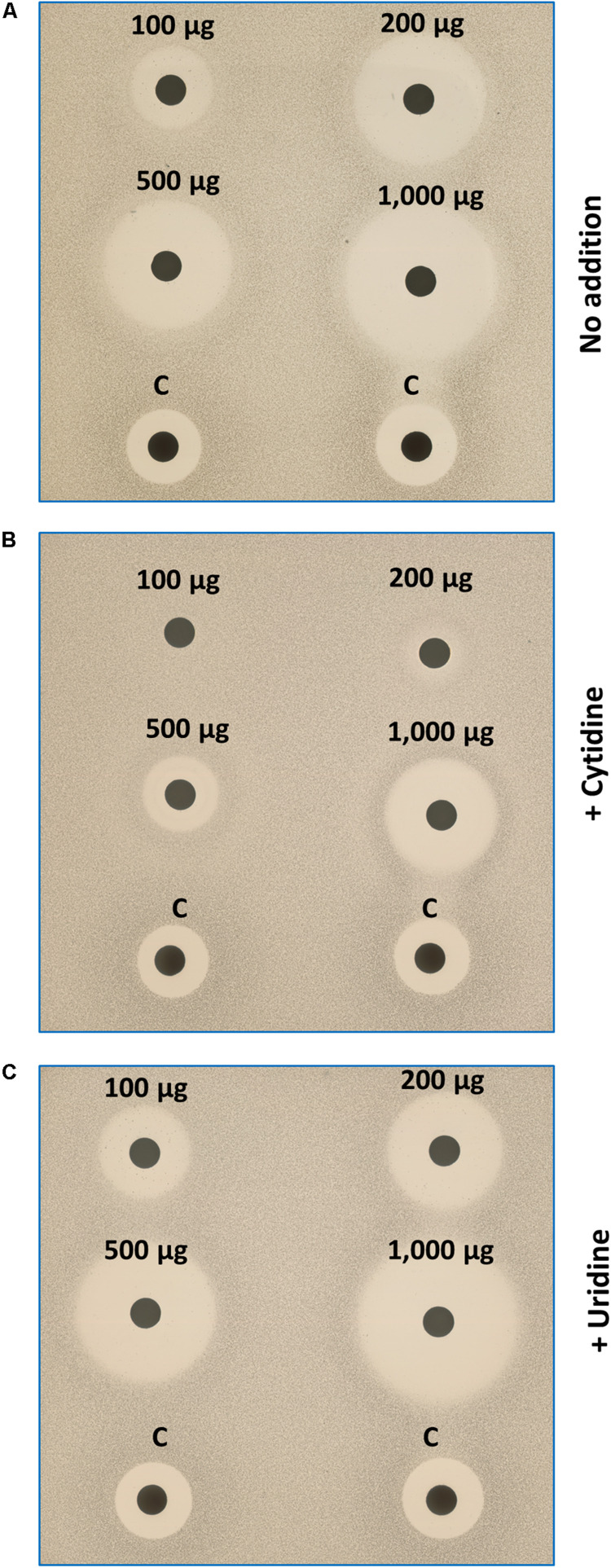
Effects of exogenous cytidine and uridine on growth of IQC treated cells. The disc diffusion plates were seeded with the *Δ4* cells. Discs were impregnated with different quantities of IQC or the control compound (Citrox). **(A)** Medium (NA/Mg^2+^) only; **(B)** Medium supplemented with cytidine (2.5 mM); **(C)** Medium with uridine supplement. The addition of cytidine (but not uridine), can compensate the CTPS inhibition by IQC.

The above results suggested that IQC inhibits growth by limiting the synthesis of CTP, which is needed for many cellular processes including RNA, DNA and fatty acid synthesis. *B. subtilis* cells can take up and incorporate nucleosides, including both cytidine and uridine (Kloudova and Fucik, 1974). If CTPS was the only or main target for IQC activity, growth in the presence of the compound might be rescued by supplementation of the culture medium with cytidine. As shown in Figure 5, the zones of inhibition generated by IQC discs were greatly diminished by the presence of 2.5 mM cytidine (Panel B), whereas uridine had no discernible effect (Panel C). This result lent strong support to the notion that the major effect of IQC is directly on the activity of CTPS.

## Discussion

### Unexpected Effects of CTPS Inhibitors on Peptidoglycan-GTase-Deficient Strains

We set out to find inhibitors of the SEDS proteins RodA and or FtsW, based on the expectation that strains lacking all four class A PBPs, which lack the alternative GTase for PG synthesis, should be more susceptible to GTase inhibitors (Emami et al., 2017). A search of a small set of compounds related to a candidate natural product inhibitor resulted in identification of the closely related IQC and IQC-3 small molecules, which were both more active on the *ΔponA* or *Δ4* strains than the wild type. Although microscopic examination of treated cells revealed a cell division deficiency, as expected for an FtsW inhibitor, further studies revealed that the division defect was likely secondary to abnormalities in chromosome replication, segregation or organization. Also, both compounds induced a DNA damage reporter, consistent with an upstream effect on chromosome dynamics. The finding that the compounds were probably acting to inhibit CTPS provided a possible explanation for the chromosome effect, since depletion of CTP would be expected to perturb dCTP levels required for DNA replication. Induction of the *liaI* reporter was also observed, indicative of some kind of cell envelope stress response. Although this could again have been due to direct inhibition of SEDS function, it could also be due to depletion of intermediates in lipid II, phospholipid or teichoic acid synthesis, all of which have pathway components involving CTP carriers. It is also possible that the extra sensitivity is the consequence of altered cell wall synthesis, which might explain why such compounds were not discovered in previous screens for CTPS inhibitors. We are presently carrying out experiments to try and understand why inhibition of CTPS is more toxic to *ponA* and *Δ4* mutants than wild type cells.

### CTPS as a Therapeutic Target

CTPS occupies a central position in intermediary metabolism and interacts with multiple key metabolites including ATP, GTP, CTP, UTP and glutamine, with ADP and glutamate (as well as CTP) being the products (reviewed by Endrizzi et al., 2004). Not surprisingly, the *pyrG* gene encoding CTPS has been shown to be essential in several different organisms (e.g., Kobayashi et al., 2003; Mori et al., 2015). CTPS has indeed been explored as a potential therapeutic target for a number of infections, especially *Mycobacterium tuberculosis* (Mori et al., 2015; Esposito et al., 2017; Chiarelli et al., 2018), but also for protozoan infections including trypanosomiasis (Fijolek et al., 2007). Nucleoside analogs acting on CTPS have also been used in human cancer therapies e.g., gemcitabine (Mccluskey et al., 2016); but these tend to have complex off target effects as they can interfere with multiple nucleotide binding proteins and even act as substrates.

To be useful as an antibiotic target the protein needs to be either missing in humans or sufficiently different to enable specific inhibitors of the pathogen protein to be isolated. There are now several structures of bacterial and human CTPS enzymes (Endrizzi et al., 2004; Mori et al., 2015; Lynch et al., 2017). These structures and complementary biochemical studies have revealed a complex higher order levels of CTPS organization. The protein has a very complex tertiary structure. The active form of the enzyme is at least a tetramer, and tetramers can then polymerize to form filamentous structures (Levitzki et al., 1971; Barry et al., 2014; Lynch et al., 2017). Polymerization appears to be a mechanism for allosteric control of enzyme activity. Remarkably, however, the effects of polymerization are completely opposite in the human and bacterial enzymes: thus, polymers of the human protein have increased activity (Lynch et al., 2017) whereas they are inactive in bacteria (Barry et al., 2014). This offers the opportunity, at least in principle, to achieve the desired specificity of inhibition in a potential therapeutic.

### IQC as a Possible Therapeutic Starting Point

IQC and IQC-3 (MW 173 Da) are small molecules with relatively high MIC values. However, the experiments described above demonstrate that despite their simplicity, they have good specificity for the CTPS enzyme, which is not displayed by closely related molecules. Several lines of evidence show that the primary if not exclusive target for these compounds is CTPS. First, the mapping to *pyrG* of resistance mutations largely overcoming the effects of the inhibitors on growth of *B. subtilis*. Second, direct inhibition of purified CTPS *in vitro*. Third, partial rescue of growth inhibition by exogenous cytidine but not the closely related nucleoside uridine. At this stage we do not know how these molecules inhibit CTPS function. IQC does not resemble any of the key substrates (ATP, UTP or glutamine), nor the allosteric regulators CTP or GTP. Thus, structural studies will be required to establish where it binds and how it affects enzyme activity. Interestingly, some of the mutations generating resistance to IQC affected amino acid residues (E156 and R159 in *B. subtilis*) that are equivalent to those found for other diverse compounds affecting bacterial, yeast or mammalian CTPS (Whelan et al., 1993; Ostrander et al., 1998; Endrizzi et al., 2005). These substitutions, and S201I, lie in the conserved N-terminal amidoligase domain, whereas P485L lies in the C-terminal glutamine amidotransferase domain at an important inter-subunit interface that seems to be involved in allosteric regulation of the protein in response to CTP binding (Endrizzi et al., 2005), so they probably work by deregulation of enzyme activity. Further structural and biochemical studies are warranted to resolve these interesting findings. Work to identify analogs of IQC with improved antibiotic properties are underway.

## Materials and Methods

### Chemicals and Media

Nucleoside triphosphates were from Thermo Fischer Scientific. All other chemicals, unless stated otherwise were from Merck.

Nutrient agar (NA, Oxoid) was used for routine selection and maintenance of both *B. subtilis* and *E. coli* strains. Liquid medium used for *B. subtilis* growth was Luria–Bertani (LB) supplemented with Mg^2+^ to a final concentration of 20 mM.

### *B. subtilis* Transformation

*Bacillus subtilis* cells were made competent for transformation with DNA either by the method of Hamoen et al. (2002) or that of Anagnostopoulos and Spizizen (1961) as modified by Jenkinson (1983).

### Minimum Inhibitory Concentration (MIC) Estimation

Luria–Bertani medium supplemented with Mg^2+^ was inoculated with one single colony and incubated at 30°C overnight. Next morning the overnight culture was diluted (1 in 1000) in fresh LB supplemented with Mg^2+^ and grown to exponential phase. The culture was diluted again in the same medium to an OD 600 nm of 0.04, then aliquoted into 96-well plates. Cells were grown for 1 h and then different concentrations of IQC, ranging from 2000 to 15 μg mL^–1^, were added to the wells using a multi-channel pipette, to a final volume of 200 μL. The growth of the cultures were monitored for 10 h at 30°C. The lowest concentration that stopped the visible growth of each strain was indicated as the MIC for the strain.

### Preparation of Genomic DNA for PCR and Genome Sequencing and Analysis

Genomic DNA was prepared using GenElute Bacterial Genomic DNA kit (SIGMA-ALDRICH). Prepared material was then passed through a 0.45 μm filter to remove any possible spores escaped during the procedure. Genomic DNA sequences of *B. subtilis* strains were performed by NU-OMICS DNA sequencing research facility, Northumbria University, Newcastle upon Tyne, using the Illumina NexteraXT technology. Briefly, an Illumina NexteraXT kit was used to create the sequencing libraries from 50 ng of genomic DNA, adding sequencing adaptors by transposition. Molecular equivalents of each library were loaded and sequenced with an Illumina MiSeq V3instrument, giving 300 base, paired end reads.

Genome *de novo* assembly and analyses were carried out using QIAGEN CLC Main Workbench software version 11.0.2 (CLC Bio, Qiagen, Valencia, CA, United States). In brief, in General methods, the “Paired reads Import” option was selected to import the Illumina sequences. The relevant pairs of each sequence were selected, then the *De Novo* Sequencing and *De Novo* Assembly path was followed. The contig sequences were generated using Map reads back to contigs. The reference genome of *Bacillus subtilis* 168 (GenBank: AL009126.3) was imported from Genbank, using the Standard Import command. Then in Toolbox, Track tools and Convert to tracks were selected. In the Toolbox, NGS core tools then Map reads to reference were selected. The removed failed reads parameter was applied. A track list of the reference genome and coding domains were generated and the genomes of the mutant strains were compared to the reference.

### Bacterial Strains

Bacterial strains used in this study are listed in Supplementary Table S1.

### Oligonucleotides

All oligonucleotides were purchased from Eurogentec Ltd and are listed in Supplementary Table S2.

### Disc Diffusion Assay for Antibiotics Sensitivity

Exponentially growing cultures were diluted to OD_600_ 0.02 then mixed with melt nutrient agar being poured into Petri dishes. Whatman^®^ 6 mm Antibiotic Assay Discs (cat# WHA2017006) containing the indicated amounts of antibiotics (as shown in the relevant figures) were put on the solidified agar containing bacteria. The plates were incubated at 30°C overnight.

Inhibition zone was measured as the radial distance from the external edge of the disc to the edge of the inhibitory zone.

### Re-creation of the IQC Resistant Mutants

DNA fragments containing the *pyrG* gene region were obtained by PCR from each IQC resistant *pyrG* mutant using primers PyrG-F1 and PyrG-R2 and Q5^®^ High-Fidelity DNA Polymerase (New England Biolabs Inc.). The PCR products, extending from 1570 bp upstream to 2247 bp downstream of *pyrG*, were then used to transform competent *Δ4* cells, with NA plates supplemented with 20 mM Mg^2+^ and containing 200 μg mL^–1^ IQC for selection.

### Reporter Strains

A disc diffusion assay was performed with the *B. subtilis* strains carrying the *lacZ* reporter gene coupled to promoters sensitive to various antibiotic activities including: *gyrA, yvgS, yjaX, ypuA, yheH*, *Φ105*, *helD*, and *liaI* (Fischer et al., 2004; Mascher et al., 2004; Urban et al., 2007). Cells were mixed with melting nutrient agar containing 100 μg mL^–1^ X-Gal before being added to Petri dishes. Effects of various compounds on induction of *lacZ* expression in strains containing reporter constructs for various cell damaging agents. Blue halos around zones of growth inhibition indicate induction of *lacZ* expression and hydrolysis of the indicator X-Gal. Each plate also contained a disc with a positive control.

### Purification of His-SUMO Tagged CTPS

#### Construction of pSF-WT14-pyrG and Derivatives

Plasmid pSF14 (curtesy of Prof. H. Murray), harboring a 14× His-SUMO domain was used as the backbone to construct the expression plasmid for the wild type PyrG (CTPS). Primers oKE-21 and oKE-22 (Supplementary Table S2) were used to generate the *pyrG* ORF with pSF14 overhangs, using *Δ4* genomic DNA as the template; primers oKE-23 and 24, with an overhang containing the *pyrG* stop or start codon, respectively, were used to amplify the plasmid backbone (Supplementary Table S2). The PCR products were then assembled using Gibson Assembly Cloning kit (New England Biolabs Inc.). After cloning of the WT *pyrG* gene, each resistant mutation was introduced into the wild type construct (pSF14-WTPyrG) by site-directed mutagenesis, using the primers specified in Supplementary Table S2.

### Purification of bdSENP1 (SUMO Protease)

*E. coli* BL21 carrying plasmid pSF1389 encoding His14-TEV-bdSENP1 (Frey and Gorlich, 2014) was grown at 37°C in 400 mL LB to an OD_600_ of 1.0. Expression of SUMO protease was induced by adding IPTG to a final concentration of 1 mM and growing at 30°C for 4 hrs. Cells were harvested by centrifugation at 4000× *g* for 15 min and re-suspended in 10 mL of Ni^2+^Binding Buffer A (250 mM NaCl, 40 mM Tris–HCl pH7.5, 10 mM MgCl2, 10 mM imidazole), containing protease inhibitor (Complete, EDTA free protease inhibitor cocktail, Roche). Cells were disrupted by sonication for 10 min at 70 W in 2−s on/4-s off pulses using a Fisherbrand FB-505 sonicator (Fisher Scientific), and cell debris was removed by centrifugation at 30,000× *g*, 4°C for 45 min. The supernatant was further clarified by filtration (0.20 μm). The following purification procedures were performed at 4°C. The clarified lysate was applied at 1 mL min^–1^ to a 5 mL His-Trap HP column (GE Healthcare) equilibrated with Ni^2+^ binding buffer A.

After several stepped washes of the column with buffer A containing 20 to 150 mM imidazole, the SUMO protease was eluted with Ni^2+^ elution Buffer B (290 mM NaCl, 45 mM Tris–HCl pH7.5, 4.5 mM MgCl2, 10 mM DTT, 500 mM imidazole). The eluent was dialysed into Buffer B with no imidazole for further use.

### Expression and Purification of Recombinant CTPS

*E. coli* BL21 was used as the expression host strain. The expression and purification of the proteins were carried out using standard protocols. Transformants harboring the overexpression plasmids were grown overnight at 37°C in LB broth containing 100 μg mL^–1^ ampicillin. The overnight cultures were diluted in fresh LB medium containing ampicillin (1:1000 V/V) and grown to an OD_600_ of 0.6, before being induced with 1 mM IPTG and incubated for 4 h at 30°C. Cell pellets were resuspended in extraction Buffer A containing protease inhibitor cocktail. Clarified lysate was passed through a 0.2 μm filter prior to loading onto a 5 mL His-Trap HP chromatography column. The column was subjected to stepped washes with buffer A containing a range of imidazole concentrations from 20 to 200 mM imidazole (5 column volume each). The protein was eluted with buffer A, containing 300 mM imidazole. The elute was then dialysed overnight at 4°C against buffer A without imidazole. The purified fusion protein was subjected to digestion with bdSENP1 (SUMO Protease) at 4°C for 48 h. The mixture was then loaded onto a His-Trap HP column freshly calibrated with Buffer A. Then the column was washed with buffer A resulting in binding of the His tagged bdSENP1 in the column and elution of un-tagged CTPS. The purity of the CTPS proteins was verified by SDS-PAGE.

### MALDI-ToF Mass Spectrometry

A RapifleX MALDI TOF/TOF mass spectrometer (Bruker Daltonics) equipped with a Smartbeam 3D laser was used in negative ion mode with Compass 2.0 control for all data acquisition. Samples were run in automatic mode (AutoXecute; Bruker Daltonics), acquiring 5000 shots at a 10-kHz frequency per spot in a random walk on spot.

Ionization was achieved using a fixed laser power of 70% (laser attenuator offset 7%, range 30%) and detected by the FlashDetector (Bruker, Bremen, Germany) at a detector gain of × 2 in the range of 100 to 2000 mass to charge ratios (*m/z*). Samples were analyzed in reflector mode with optimized voltages for reflector 1 (20.82 kV), reflector 2 (1.085 kV), and reflector 3 (8.8 kV), ion sources (ion source 1, 20 kV, PIE 2.66 kV), lens (11.3 kV), and a pulsed ion extraction of 200 ns. Spectra were accumulated with FlexControl software (v4.0) and processed using FlexAnalysis software (v4.0) (Bruker Daltonics). Calibration was performed using UTP, CTP, and GTP (Thermo Fisher). Fresh suspension of 10 mg mL^–1^ of 9-aminoacridine in 10% methanol was prepared, then centrifuged and the supernatant was used in a 1:1 ratio (v/v) with each sample on a MTP polished steel plate. The spotted targets were allowed to air dry before MALDI-MS analysis.

### CTP Synthetase Assays and Kinetic Analysis

CTP synthetase activity was determined by measuring the conversion of UTP to CTP (molar extinction coefficients of 182 and 1520 M21 cm-1, respectively (Long and Pardee, 1967) as an increase in absorbance at 291 nm (Nadkarni et al., 1995). The absorbance was monitored using a SpectroStra Nano (BMG LABTECH). Where required, the concentration of CTP was calculated using *A*_29__1_ = ε (1338 M^–1^ cm^–1^) x c (mol L^–1^) × L (1.03 cm) equation (Habrian et al., 2016). The standard reaction mixture contained 50 mM Tris–HCl, pH 7.5, 10 mM MgCl2, 1 mM DTT, 2 mM L-glutamine, 0.1 mM GTP, 2 mM ATP, 2 mM UTP, and an appropriate dilution of enzyme protein in a total volume of 0.2 mL. The initial rates were extracted from the earliest linear regions of *A*_29__1_ versus time data using linear regressions.

The kinetic parameters *V*_*max*_ and *K*_*m*_ were determined by running a series of 8 h CTPS assay at UTP concentrations ranging from 0 to 6 mM. The parameters were assessed by plotting the reaction against UTP concentration. The ligand-binding option, and then Michaelis−Menten and Hill equation were applied using SigmaPlot 13.1 (Systat Software Inc.). The values were determined in triplicate and the mean values are reported in Figure 4. The reported errors are ± Standard Deviation (SD).

### IC_50_ Determination by Glutamate Assay

Glutamate assay kit was purchased from SIGMA ALDRICH (cat # MAK004) and the supplier’s protocol was followed to measure the amount of glutamate released by purified CTP synthetase species. The enzymatic reaction was set up as described before. The reaction was stopped by boiling the samples for 10 min, before adding the reaction components for development of the color and reading the absorbance at 450 nm using a BMG SpectroStra Nano (BMG LABTECH). The estimated IC_50_ values were established by sigmoidal non-linear regression, 4-parameter logistic function, using Sigma Plot 13.1 software.

### Microscopic Imaging

Overnight cultures, grown in LB supplemented with Mg^2+^ (20 mM) at 30°C, were diluted (1:1000 V/V) into fresh medium and grown at 30°C until exponential phase. The exponentially growing cultures were then diluted again to an OD of 0.02, and IQC was added to a final concentration of 150 or 300 μg mL^–1^. For “no IQC” control, DMSO was added to a final concentration of 0.01%. Growth of the cultures was continued at 30°C. For microscopy 2 μl of cells were mounted onto microscope slides coated with a thin film of 1.2% agarose in water. To visualize membranes and DNA, 30 μl culture was mixed with 1 μl of membrane dye FM5-95 (200 μg ml^–1^; Molecular Probes) then, if used, with 3 μl DNA dye 4,6-diamidino-2-phenylindole (DAPI; Sigma; 1 μg ml^–1^ in 50% glycerol), before adding onto the agarose slide. Images were acquired with a Rolera EM-C2 (Q-imaging) camera attached to a Nikon Ti microscope using METAMORPH version 6 (Molecular Devices). Images were processed using Fiji (NIH).

## Data Availability Statement

All datasets presented in this study are included in the article/Supplementary Material.

## Author Contributions

KE and LW did the experiments. All authors contributed to experimental design and concepts. JE wrote the main text with contributions from KE and LW.

## Conflict of Interest

The authors declare that the research was conducted in the absence of any commercial or financial relationships that could be construed as a potential conflict of interest.
