# Supplementary figures and images for: A Small Molecule Inhibitor of CTP Synthetase Identified by Differential Activity on a Bacillus subtilis Mutant Deficient in Class A Penicillin-Binding Proteins
Source: Front Microbiol. 2020 Aug 26;11:2001. doi: 10.3389/fmicb.2020.02001 (PMC7479849; doi:10.3389/fmicb.2020.02001)

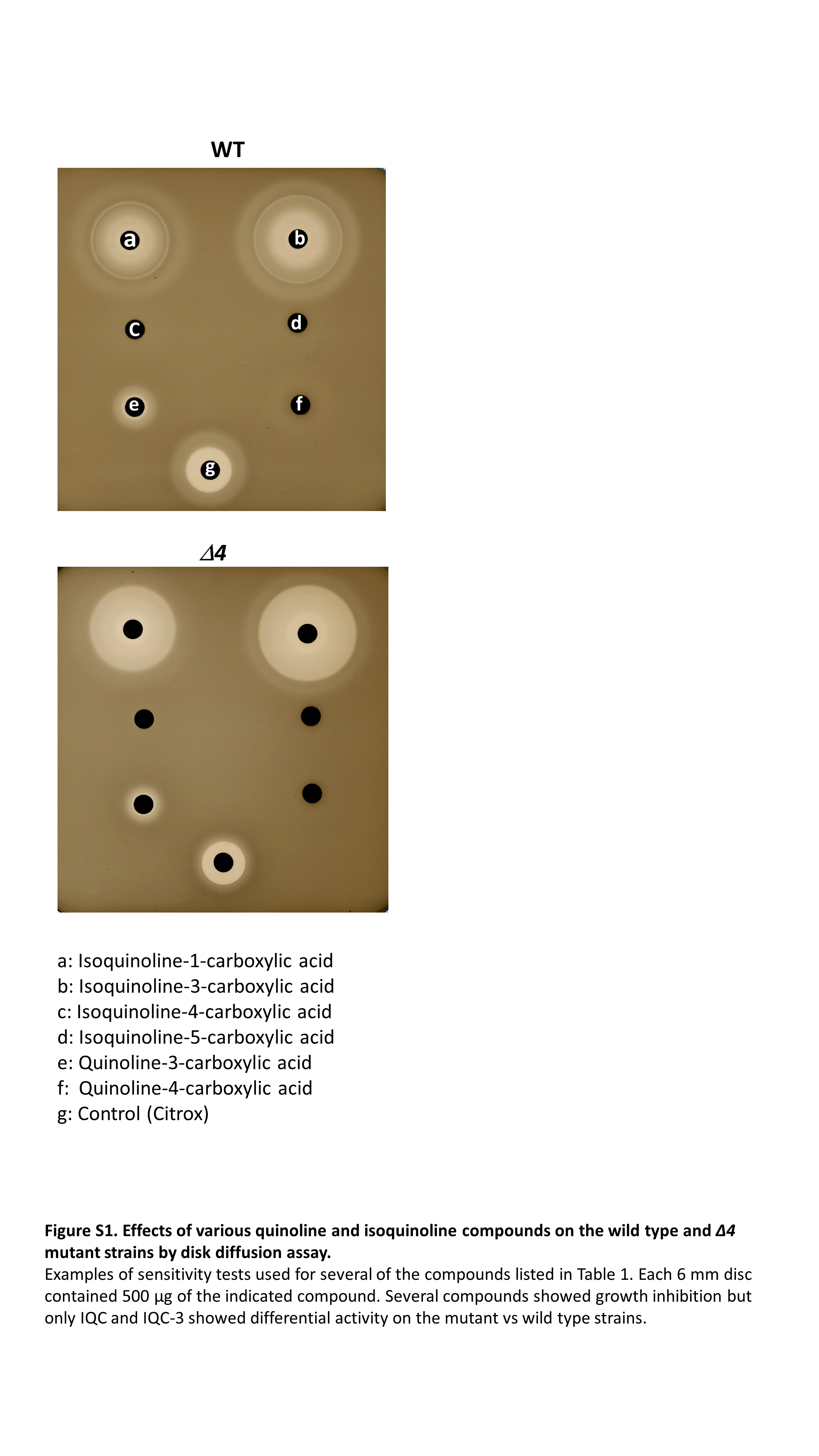

Supplement: Supplementary file 1 [file Image_1.tif]

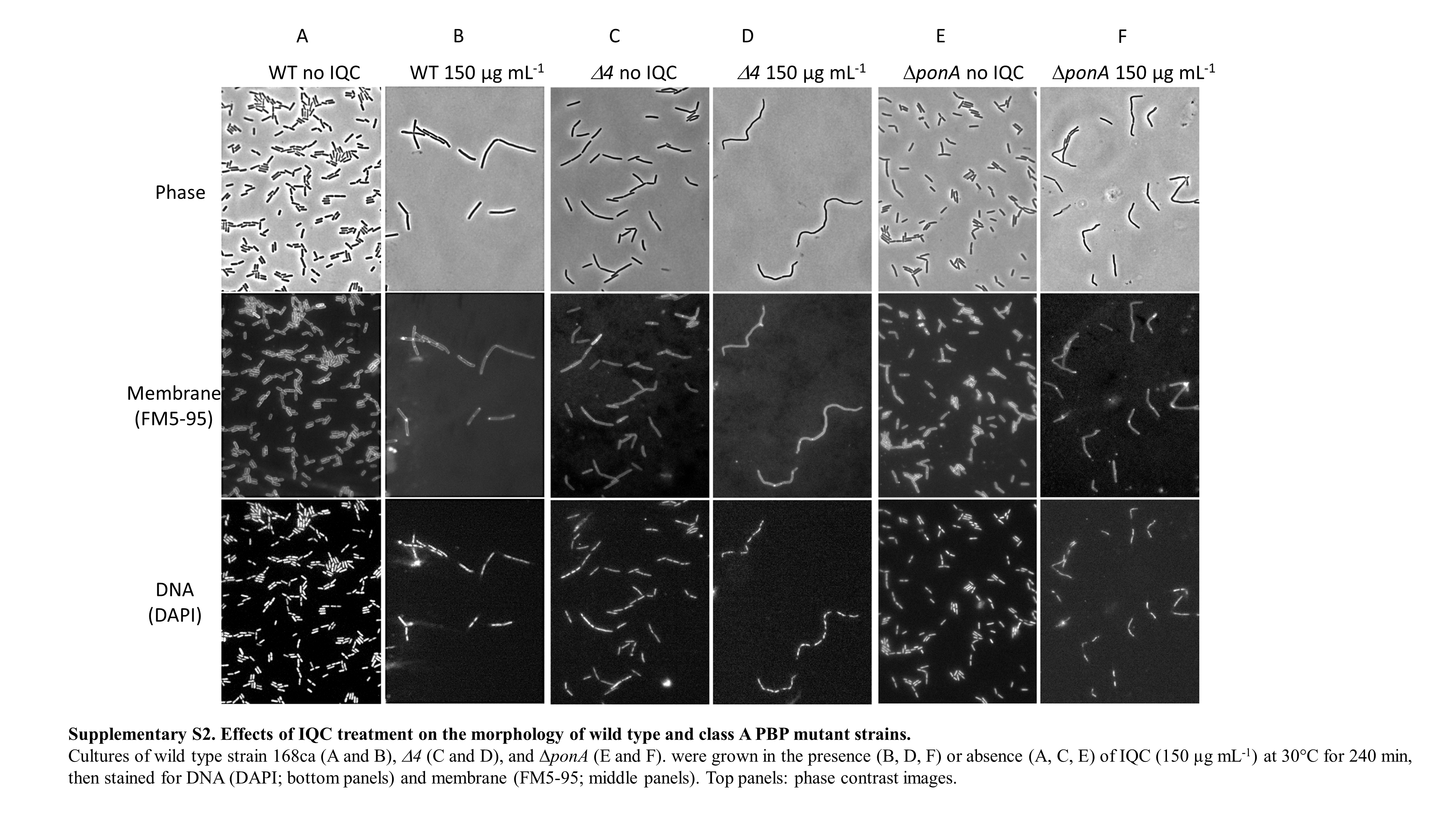

Supplement: Supplementary file 2 [file Image_2.tif]

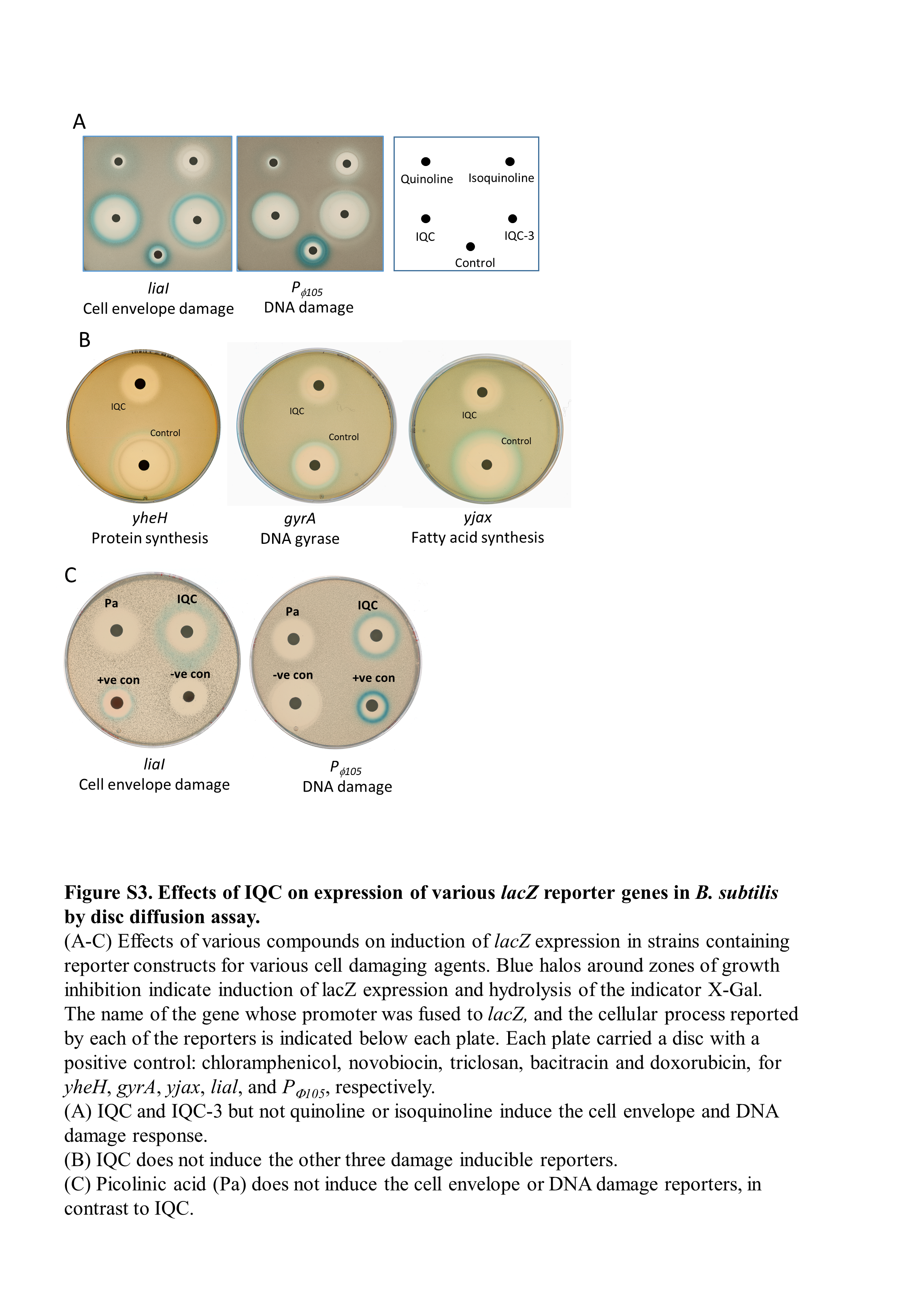

Supplement: Supplementary file 3 [file Image_3.tif]

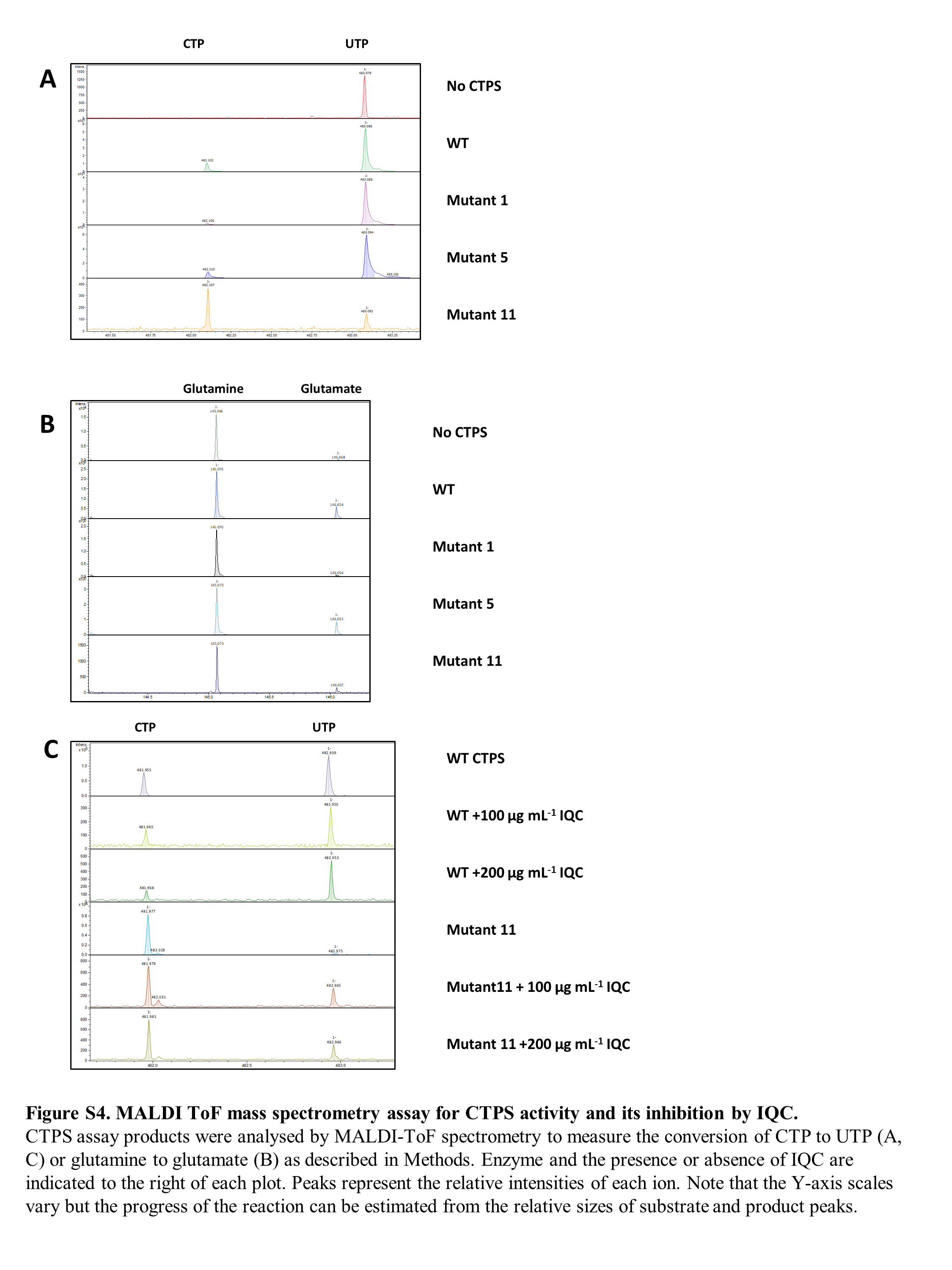

Supplement: Supplementary file 4 [file Image_4.tif]
