# Supplementary material for: A Small Molecule Inhibitor of CTP Synthetase Identified by Differential Activity on a Bacillus subtilis Mutant Deficient in Class A Penicillin-Binding Proteins
Source: Front Microbiol. 2020 Aug 26;11:2001. doi: 10.3389/fmicb.2020.02001 (PMC7479849; doi:10.3389/fmicb.2020.02001)
Supplement: Supplementary file 5 [file Table_1.pdf]

Table S1- Bacterial strains used in this study

Strains	Source/Genotype	References/cat #
E. coli DH5α	F- Φ80*lacZΔM15 Δ(lacZYAargF)U196 recA1 endA1 hsdR17 (rK-, mK+) phoA supE44 λ- thi1*	CBCB stock
E. coli BL21 (DE3)	Novagen	Cat# 69450
168ca	*trpC2*	Lab stock
RE101	168ca *ΔponA::spec*	Emami et al. (2017)
AG157	168ca *ΔponA ΔpbpD ΔpbpF ΔpbpG::kan*	Emami et al. (2017)
EzrA-GFP	*Ezra::PezrA-gfp::cat*	Gamba et al. (2009)
168ca hbsU-mCherry-neo	*amyE*::*neo hbsU-gfp*	168 hbsU-mCherry-cat (JW Veening, unpubl) converted to neo-resistant.
