# Supplementary material for: A Small Molecule Inhibitor of CTP Synthetase Identified by Differential Activity on a Bacillus subtilis Mutant Deficient in Class A Penicillin-Binding Proteins
Source: Front Microbiol. 2020 Aug 26;11:2001. doi: 10.3389/fmicb.2020.02001 (PMC7479849; doi:10.3389/fmicb.2020.02001)
Supplement: Supplementary file 6 [file Table_2.pdf]

Table S2-Sequence of the oligonucleotides used in this work

Oligo’s ID	Sequence (5′> 3′)	Description
PyrG-F1	CGATCCCGAGAAAAAGAAAAAAGG	To amplify *pyrG* ORF + 1570 bp upstream sequence
PyrG-R2	GTTTTCCGTCTATCTTTAGAAAG	To amplify *pyrG* ORF + 2247 bp extra downstream sequence
oKE-21	CCATCACCATATGACGAAATATATTTTT GTAACCGGGG	*pyrG* + pSF14 overhangs (11 nucleotides)
oKE-22	CAGCCGGATCTTACTTCTGATTTGCAGC TTCGACAG	*pyrG* + pSF14 overhangs (10 nucleotides)
oKE-23	CAAATCAGAAGTAAGATCCGGCTGCTAA CAAAGCC	pSF14 Fw + *pyrG* 11 nucleotides overhangs including the stop codon
oKE-24	TATATTTCGTCATATGGTGATGGCCTGA ATGATGG	pSF14 Rv +14x His + pyrG 13 nucleotides overhangs, including the start codon
oKE-29-Pyr-Mut1F	CGAATCACTGCCATTCCTTGGAGCGATC	Site directed mutagenesis-Mutant 1
oKE-30-Pyr-Mut1R	GCTCTTCATTTGGCGGATCGCTCCAAG	Site directed mutagenesis-Mutant 1
oKE-31-Pyr-Mut11F	CCATTCCTTGAAGCGATCTGCCAAATG	Site directed mutagenesis-Mutant 11
oKE-32-Pyr-Mut11R	CCGATGTCGCTCTTCATTTGGCAGATC	Site directed mutagenesis-Mutant 11
oKE-35-Pyr-Mut5F	GCCCTGATGGACGCCTTGTTG	Site directed mutagenesis-Mutant 5
oKE-36-Pyr-Mut5R	GTCCATCAGGGCTTGTGCCTGAG	Site directed mutagenesis-Mutant 5
oKE-37-PyrG-F	GACGAAATATATTTTTGTAACCGGGG	To check mutation sites in *PyrG*
okE38-PyrG-R	CTTACTTCTGATTTGCAGCTTCGACAGA C	To check mutation sites in *PyrG*
oKE39-pSF14 -F	CCGTCAGTCTGTAGACATGAC	To check the sequence of *pyrG*
